# Typhoid fever presenting with gastric ulcer bleeding

**DOI:** 10.1186/s12876-022-02192-2

**Published:** 2022-03-10

**Authors:** Hong Jae Jeon, Jong Seo Lee, Byung Seok Lee, Seok Hyun Kim, Eaum Seok Lee, Jae Kyu Sung, Hee Seok Moon, Sun Hyung Kang, Hyun Seok Lee, Seongwoo Choi, Heon Sa-Kong, Shinhye Cheon, Hyuk Soo Eun

**Affiliations:** 1 Division of Gastroenterology, Department of Internal Medicine, Chungnam National University Hospital, 282, Munhwa-ro, Jung-gu, Daejeon, 35015 Republic of Korea; 2 Division of Infectious Disease, Department of Internal Medicine, Chungnam National University Hospital, 282, Munhwa-ro, Jung-gu, Daejeon, 35015 Republic of Korea; 3 Department of Internal Medicine, School of Medicine, Chungnam National University, 282, Munhwa-ro, Jung-gu, Daejeon, 35015 Republic of Korea

**Keywords:** Typhoid fevers, *Salmonellaenterica* serovar Typhi, Gastrointestinal hemorrhages, Gastric ulcer, Case report

## Abstract

**Background:**

Enteric fever is a systemic disease caused by *Salmonella enterica* serovar Typhi or *Salmonella enterica* serovar Paratyphi, characterized by high fever and abdominal pain. Most patients with enteric fever improve within a few days after antibiotic treatment. However, some patients do not recover as easily and develop fatal life-threatening complications, including intestinal hemorrhage. Lower gastrointestinal bleeding has been reported in 10% of cases. However, upper gastrointestinal bleeding has rarely been reported in patients with enteric fever. We present a case of gastric ulcer hemorrhage caused by enteric fever.

**Case presentation:**

A 32-year-old woman, complaining of fever lasting four days and right upper quadrant pain and melena that started one day before admission, consulted our hospital. Abdominal computed tomography revealed mild hepatomegaly and gastroscopy revealed multiple active gastric ulcers with flat black hemorrhagic spots. The melena of the patient stopped on the third day. On the fifth admission day, she developed hematochezia. At that time, *Salmonella enterica* serovar Typhi was isolated from the blood culture. The antibiotic regimen was switched to ceftriaxone. Her hematochezia spontaneously resolved the following day. Finally, the patient was discharged on the 12th admission day without clinical symptoms. However, her fever recurred one month after discharge, and she was readmitted and *Salmonella enterica* serovar Typhi was confirmed again via blood culture. She was treated with ceftriaxone for one month, and was discharged without complications.

**Conclusion:**

Our case showed that although rare, active gastric ulcers can develop in patients with enteric fever. Therefore, upper and lower gastrointestinal bleeding should be suspected in patients with enteric fever, especially showing relapsing bacteremia.

## Background

Enteric fever is a systemic disease caused by *Salmonella enterica* serovar Typhi or *Salmonella enterica* serovar Paratyphi. The bacteria usually invade the small intestinal mucosa, where they infect and multiply via the lymphatic/blood vessels. Typical symptoms include febrile symptom, whole abdominal pain and tenderness, diarrhea, anorexia, and weight loss [1, 2]. Most people diagnosed with enteric fever improve a few days after empirical antibiotic treatment. However, some patients develop complications [1, 3, 4]. The complications of enteric fever can affect any organ system. The important gastrointestinal manifestations of enteric fever include hepatosplenomegaly, jaundice, lower gastrointestinal hemorrhage, intestinal perforation, and acalculous cholecystitis [5]. Intestinal hemorrhage or perforation is the most serious complication of enteric fever [5–7]. Occurring in 10% of cases, lower gastrointestinal bleeding most commonly affects the terminal ileum and ileocecal valve [5, 8]. Meanwhile, upper gastrointestinal bleeding has rarely been reported. We present a patient with enteric fever, presenting with gastric ulcer hemorrhage, as a rare complication of disease.

## Case presentation

**Fig. 1:**
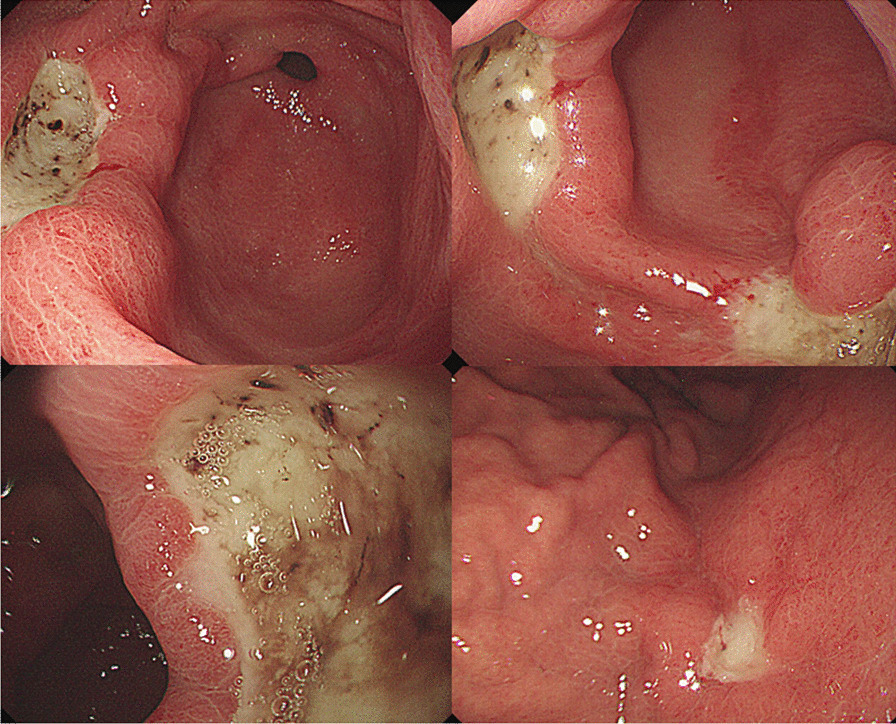
Esophagogastroduodenoscopy revealed three gastric ulcers with flat black hemorrhagic spots, which were located from the anterior and posterior walls of the gastric antrum to the greater curvature of the gastric midbody

**Fig. 2:**
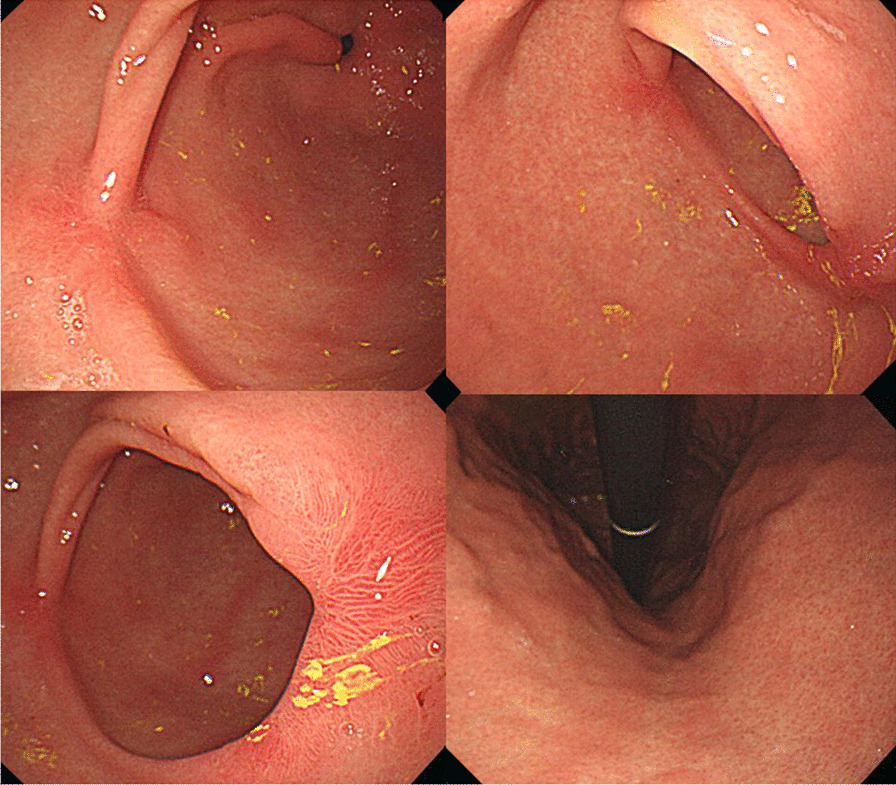
Follow-up esophagogastroduodenoscopy revealed a significantly healed gastric ulcer

**Fig. 3:**
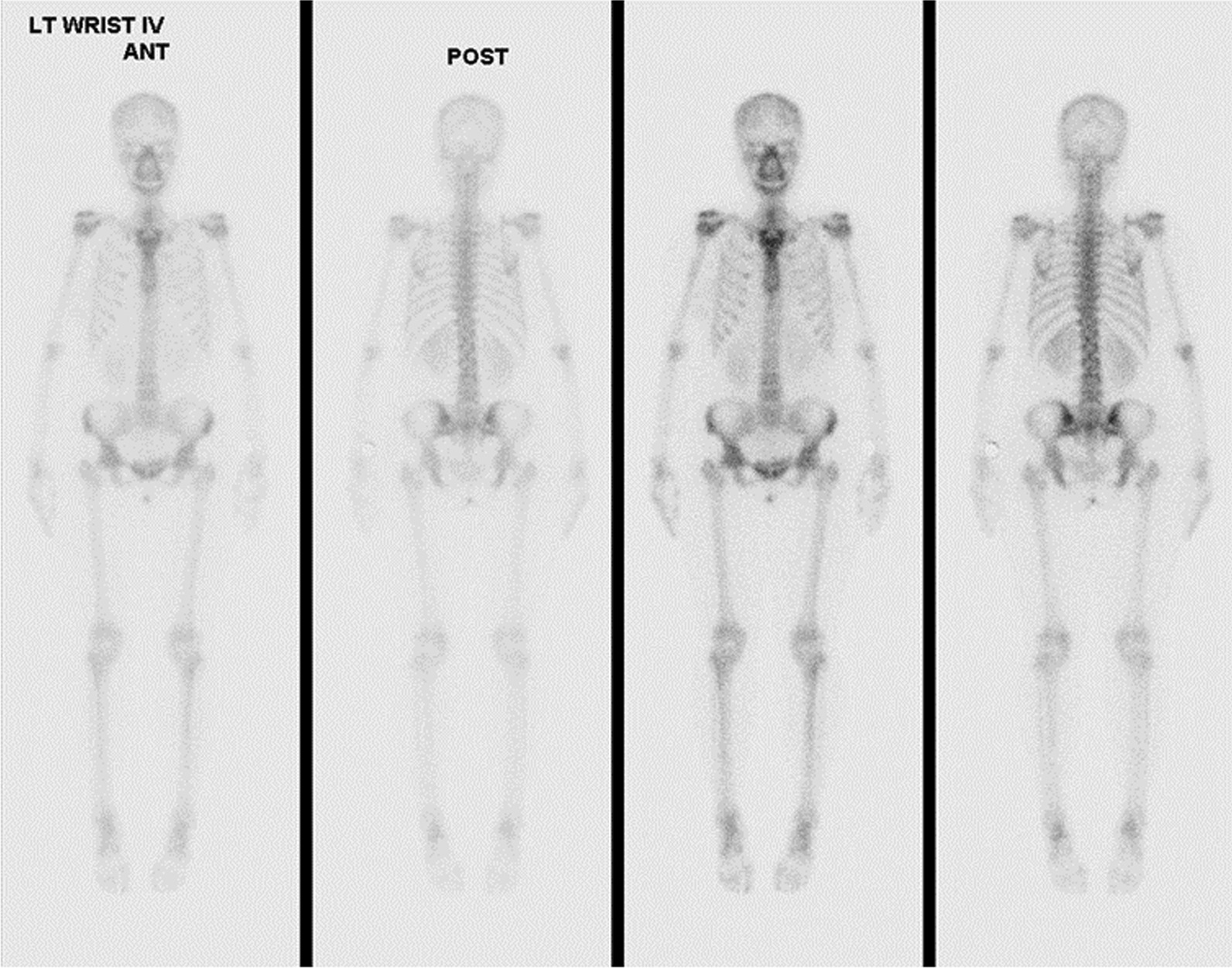
Whole body bone scan showed no obvious uptake

**Fig. 4:**
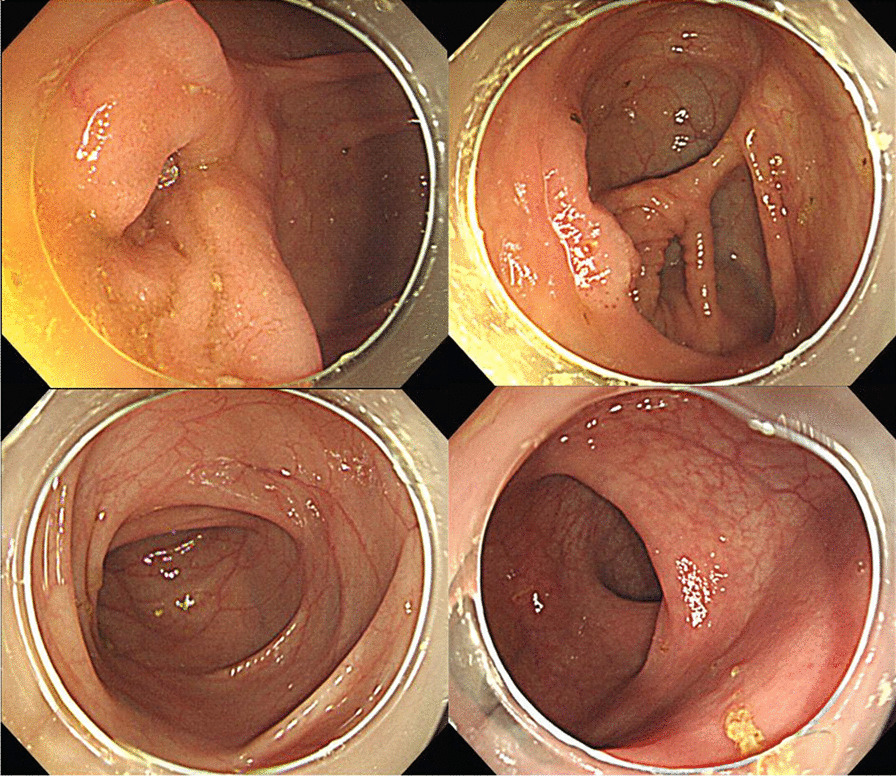
No lesions were seen at colonoscopy

A 32-year-old woman consulted our hospital for fever lasting four days, and right upper quadrant pain and melena that started the day before admission. Her medical and family histories were unremarkable. She did not drink alcohol and was a nonsmoker. Physical examination revealed moderate right upper quadrant tenderness and a high fever (39.8 °C). Her initial laboratory workup showed an aspartate transaminase (AST) of 106 IU/L, alanine transaminase (ALT) of 97 IU/L, gamma-glutamyl transpeptidase of 108 IU/L, alkaline phosphatase of 123 IU/L, lactate dehydrogenase of 682 IU/L, and C-reactive protein of 14.2 mg/dL. Her hemoglobin level, white blood cell count, and platelet count were within normal range. The serologic markers of viral hepatitis A, B, and C were all negative. Abdominal computed tomography and magnetic resonance cholangiopancreatography revealed mild hepatomegaly and a small benign hepatic cyst measuring 0.8 cm. However, esophagogastroduodenoscopy revealed three gastric ulcers with flat black hemorrhagic spots, which were located from the anterior and posterior walls of the gastric antrum to the greater curvature of the gastric midbody (Fig. 1). The patient did not take medications, such as non-steroidal anti-inflammatory drugs, that could cause gastric ulcers. Histopathological examination of the biopsy specimen, obtained from each ulcer, revealed chronic atrophic gastritis. The patient was treated with antibiotics, hepatotonics, and proton pump inhibitor. The additional serologic tests, performed after admission, showed no evidence of Epstein-Barr virus hepatitis, cytomegalovirus hepatitis, Wilson’s disease, autoimmune hepatitis, or other rare type of hepatitis. The patient’s melena was relieved by conservative treatment on the third admission day, and her hemoglobin level was maintained at 12.5 g/dL. On the fifth day after admission, she abruptly developed hematochezia, and her hemoglobin dropped from 12.5 to 10.8 g/dL. Her AST and ALT levels increased to 442 IU/L and 248 IU/L, respectively. At a time, *Salmonella enterica* serovar Typhi was isolated from blood cultures. Upper endoscopy and colonoscopy, and liver biopsy were reserved for severe hematochezia or persistent elevation of liver enzymes. The antibiotic regimen was switched from piperacillin-tazobactam plus moxifloxacin to ceftriaxone, according to the antibiotic susceptibility test. The next day, the patient’s hematochezia stopped, and she started passing loose stools with near-normal color. Her AST and ALT levels gradually decreased, and she was discharged on the 12th day after admission without clinical symptoms. However, her fever recurred one month after her discharge, and she was readmitted and *Salmonella enterica* serovar Typhi was confirmed again via blood culture. Follow-up esophagogastroduodenoscopy (EGD) was performed after 7 weeks of initial EGD and revealed a significantly healed gastric ulcer (Fig. 2). Whole body bone scan and colonoscopy were performed to identify the reservoir for the relapsed infection, however, she had a negative result (Figs. 3, 4). She was treated with ceftriaxone for additional one month and was discharged without complications.

## Discussion and conclusions

This is a case of a 32-year-old woman who presented with melena, fever, and right upper quadrant pain, was diagnosed with enteric fever with gastric ulcer bleeding, and treated with antibiotics and proton pump inhibitor. Gastrointestinal bleeding has been reported in 10% of enteric fever patients [5]. However, most of the gastrointestinal bleedings with enteric fever reported in the literature were related to small intestine or colonic lesions. In one study, analyzing the endoscopic findings in patients with enteric fever and gastrointestinal bleeding, the most commonly affected site was the terminal ileum, followed by the ileocecal valve, ascending colon, and transverse colon. Multiple variable-sized punched-out ulcers with slightly elevated margins were representative endoscopic findings [8]. Although there are many studies on lower gastrointestinal bleeding in patients with enteric fever, upper gastrointestinal bleeding has rarely been reported in patients with enteric fever. There have been only a few reports on patients, who presented with melena, diagnosed with aortoduodenal fistula associated with nontyphoidal *Salmonella* aortitis [9, 10]. Harza et al. recently reported the case of a patient with enteric fever, who presented with hematemesis and melena, were diagnosed with primary aortoduodenal fistula due to infected aortic aneurysm with *Salmonella enterica* serovar Typhi infection, and underwent emergency endoaneurysmorrhaphy [11]. However, cases of enteric fever, presenting with gastric ulcer bleeding, have not been reported until now. To the best of our knowledge, this is the first case report of enteric fever presenting with gastric ulcer bleeding. The most common causes of peptic ulcer disease are *Helicobacter pylori* infection and ingestion of nonsteroidal anti-inflammatory drugs (NSAIDs). *Helicobacter pylori* was not identified in gastric biopsy specimens from our patient, and she had no history of taking NSAIDs or other ulcerogenic drugs. Also, she was not taking any proton pump inhibitors, antibiotics, or bismuth compounds that could cause the missed diagnosis of *Helicobacter pylori* infection. Moreover, she was young and no had underlying disease. Therefore, gastric ulcer bleeding in our patient can be presumed to be caused by *Salmonella enterica* serovar Typhi infection. Enteric fever induces various clinical symptoms by forming typhoid nodules, which indicate lymphoid hyperplasia, in the reticuloendothelial system, including the liver, spleen, and other lymphoid organs. Mucosal ulcer formation or ulcer bleeding occurring in the lower gastrointestinal tract was also associated with mucosal ulceration secondary to hyperplasia of the Peyer’s patches or lymphoid follicles. However, the mechanism behind gastric ulcer formation in the present case is unclear. Interestingly, an experimental study involving *Salmonella enterica* serovar Typhimurium-infected mice, frequently used as an animal model for enteric fever, found that *Salmonella enterica* serovar Typhi infection may induce hemorrhagic ulcers by promoting gastric oxidative stress. Ofloxacin, lysozyme chloride, and oxyradical scavengers, including glutathione, allopurinol, and dimethylsulfoxide ameliorated hemorrhagic ulcer formation [12]. Another study using rats reported that *Salmonella enterica* serovar Typhi infection may cause hemorrhagic gastric ulcers by increasing histamine secretion and vascular permeability [13].

Despite these findings, there have been no reported cases or mechanistic studies of enteric fever accompanied by gastric ulcer bleeding. Our patient, who presented with gastric ulcer bleeding, was discharged after her symptoms had improved with antibiotic treatment. However, she was readmitted due to recurrence with a positive blood *Salmonella enterica* serovar Typhi blood culture. Based on this finding, the factors associated with the relapsing enteric fever could be related to the development of gastric ulcer bleeding. Enteric fever can relapse within one month in about 5–10% of patients [3, 4]. Several studies have reported that the gallbladder or mesenteric lymph node served as niches for persisting Salmonella, but the mechanism behind relapsing enteric fever remains unclear [14, 15]. Further studies on the mechanism underlying recurrent enteric fever could explain the cause of gastric ulcer bleeding which is a rare clinical symptom of enteric fever.

In conclusion, our case suggested that, although rare, peptic ulcers could develop in patients with enteric fever. Upper gastrointestinal bleeding as well as lower gastrointestinal bleeding should be suspected in patients exhibiting symptoms suggestive of gastrointestinal bleeding, especially presenting relapsing bacteremia.

## Data Availability

All data generated during this study are included in this published article.

## Abbreviations

ALT: Alanine transaminase
AST: Aspartate transaminase
NSAIDs: Nonsteroidal anti-inflammatory drugs
