# Correction to: Typhoid fever presenting with gastric ulcer bleeding

**DOI:** 10.1186/s12876-022-02261-6

**Published:** 2022-05-10

**Authors:** Hong Jae Jeon, Jong Seo Lee, Byung Seok Lee, Seok Hyun Kim, Eaum Seok Lee, Jae Kyu Sung, Hee Seok Moon, Sun Hyung Kang, Hyun Seok Lee, Seongwoo Choi, Heon Sa-Kong, Shinhye Cheon, Hyuk Soo Eun

**Affiliations:** 1 Division of Gastroenterology, Department of Internal Medicine, Chungnam National University Sejong Hospital, 20, Bodeum 7-ro, Sejong, 30099 Republic of Korea; 2 Division of Gastroenterology, Department of Internal Medicine, Chungnam National University Hospital, 282, Munhwa-ro, Jung-gu, Daejeon, 35015 Republic of Korea; 3 Division of Infectious Disease, Department of Internal Medicine, Chungnam National University Hospital, 282, Munhwa-ro, Jung-gu, Daejeon, 35015 Republic of Korea; 4 Department of Internal Medicine, School of Medicine, Chungnam National University, 266, Munhwa-ro, Jung-gu, Daejeon, 35015 Republic of Korea

## Correction to: BMC Gastroenterology (2022) 22:116 10.1186/s12876-022-02192-2

After publication of this article [1], the authors reported that the affiliation details for the first author were incorrectly given as “Division of Gastroenterology, Department of Internal Medicine, Chungnam National University Hospital, 282, Munhwa-ro, Jung-gu, Daejeon 35015, Republic of Korea”, but should have been “Division of Gastroenterology, Department of Internal Medicine, Chungnam National University Sejong Hospital, 20, Bodeum 7-ro, Sejong 30099, Republic of Korea”.

The original article [1] has been updated.

